# Supplementary material for: Cloud BioLinux: pre-configured and on-demand bioinformatics computing for the genomics community
Source: BMC Bioinformatics. 2012 Mar 19;13:42. doi: 10.1186/1471-2105-13-42 (PMC3372431; doi:10.1186/1471-2105-13-42)
Supplement: Additional file 1 — Supplementary 1 Cloud BioLinux software documentation in the form of a mini, self-contained website. Users need to download and uncompress the .zip file, and open through a web browser the "index.html" file available on the main directory. (ZIP 1823 kb). [file 1471-2105-13-42-S1.ZIP › Cloud-BioLinux-Package-Documentation/docs/clmconf.html]

Bio-Linux Software Documentation Pages

Back to search form

## clmconf

|  |  |
| --- | --- |
| Name | clmconf |
| Description | **clmconf** allows the user to create confidence measures for individual nodes and clusters  TribeMCL is a method for clustering proteins into related groups, which are termed 'protein families'. This clustering is achieved by analysing similarity patterns between proteins in a given dataset, and using these patterns to assign proteins into related groups. In many cases, proteins in the same protein familywill have similar functional properties. TribeMCL uses a novel clustering method (Markov Clustering or MCL) which solves problems which normally hinder protein sequence clustering. These problems include: multi-domain proteins, peptide fragments and proteins which possess domains which are very widespread (promiscuous domains). The efficiency of the method makes it applicable to the clustering of very large datasets.  The algorithm is composed of the core MCL algorithm (written by Stijn Van Dongen) and the modules for biological sequence clustering written by Anton Enright. Both are written in the C language, and source code is available. |
| Homepage | http://www.ebi.ac.uk/research/cgg/tribe/ |
| Remote Documentation | http://www.ebi.ac.uk/research/cgg/tribe/manual.txt |
